# Genetic differentiation of mainland-island sheep of Greece: Implications for identifying candidate genes for long-term local adaptation

**DOI:** 10.1371/journal.pone.0257461

**Published:** 2021-09-16

**Authors:** Antonios Kominakis, Eirini Tarsani, Ariadne L. Hager-Theodorides, Ioannis Mastranestasis, Dimitra Gkelia, Ioannis Hadjigeorgiou

**Affiliations:** 1 Department of Animal Science, Agricultural University of Athens, Athens, Greece; 2 Breeder’s Association of the Lesvos sheep, Anaxos Lesvos, Greece; 3 Association of Pastoral Farmers of Epirus, Ioannina, Greece; National Cheng Kung University, TAIWAN

## Abstract

In Greece, a number of local sheep breeds are raised in a wide range of ecological niches across the country. These breeds can be used for the identification of genetic variants that contribute to local adaptation. To this end, 50k genotypes of 90 local sheep from mainland Greece (Epirus, n = 35 and Peloponnesus, n = 55) were used, as well as 147 genotypes of sheep from insular Greece (Skyros, n = 21), Lemnos, n = 36 and Lesvos, n = 90). Principal components and phylogenetic analysis along with admixture and spatial point patterns analyses suggested genetic differentiation of ‘mainland-island’ populations. Genome scans for signatures of selection and genome-wide association analysis (GWAS) pointed to one highly differentiating marker on OAR4 (F_ST_ = 0.39, FLK = 21.93, FDR p-value = 0.10) that also displayed genome wide significance (FDR p-value = 0.002) during GWAS. A total number of 6 positional candidate genes (*LOC106990429*, *ZNF804B*, *TEX47*, *STEAP4*, *SRI and ADAM22*) were identified within 500 kb flanking regions around the significant marker. In addition, two QTLs related to fat tail deposition are reported in genomic regions 800 kb downstream the significant marker. Based on gene ontology analysis and literature evidence, the identified candidate genes possess biological functions relevant to local adaptation that worth further investigation.

## Introduction

Post domestication sheep (*Ovis aries*) have spread and distributed over a wider geographic range around the globe and adapted to a variety of diverse, often harsh, environments. Following the global trend, a number of indigenous sheep breeds are found in Greece, in a range of ecological and environmental conditions [1]. Amongst them, two local breeds (Katsika and Oreino) are traditionally kept on the alpine climate zone of the Pindos mountain chain under a vertical transhumance system with livestock displacement from lowlands to highlands in summer and *vice versa*, in wintertime.

At the south western part of the country, and specifically on natural pastures of Peloponnesus’ mountains, local sheep populations prevail, with most distinctive specimens that of the rare breed known as Kokovitiko. Here the pastoral landscape is characterized by a rough terrain covered by diverse and lush vegetation due to the higher amount of rainfall than the eastern part of this peninsula. There is no transhumance practice in this area.

At the other geographical extreme of the country, and specifically on two islands of the North Aegean Sea i.e. Lemnos and Lesvos, two more breeds named after the homonymous islands, are kept in a semi-arid climate zone with low winter temperatures and longer summer droughts ([2,3]). In a similar climatic landscape, in the middle of the Aegean Sea, the sheep of Skyros have been bred in isolation for years on the homonymous island.

The above local sheep present valuable genetic resources and are critical for preserving local genetic diversity and ecosystem integrity, as well as food chain productivity and resilience. With regard to the latter, milk from these ewes is used to produce famous, high quality dairy products, of which some cheeses have been granted protected designations of origin, such as Feta in Epirus and Peloponnesus, Ladotyri in Lesvos (PDO), Kalathaki (PDO), and Melichloro in Lemnos.

Local sheep breeds traditionally raised under extreme environments are an excellent model for studying genetic adaptation [4]. Under these conditions, selective breeding for improved (re)productive performance is limited or non-existent, leaving natural selection as the primary evolutionary acting mechanism. In such cases, natural selection causes distorted patterns of genetic variation at and around selected loci, resulting in local molecular footprints that are manifested as reduced levels of genetic diversity, extended homozygosity tracts and increased differentiation from other populations [5]. Adaptation specific footprints can nowadays be detected using genome-wide data and methods for tracking specific signals in the genome (genome scans). Some scans look for alleles that are correlated with particular features of the environment. Others look for highly differentiated genomic regions between extreme populations that exceed neutral expectation, based on the notion that local adaptation will increase the frequency of locally beneficial alleles and the variation among populations in allele frequencies [6]. Regions in the genome that have been preferentially increased in frequency and fixed in a population because of their functional importance in specific processes are called signatures of selection (SS) [7] and can provide valuable information on the genetic mechanism of local adaptation and speciation [8].

In sheep, SS have been detected in a variety of wild and domestic sheep populations ([9–17]). Putative genomic regions and genes associated with breed formation [18], climate-mediated selection pressure [19] and local environmental adaptation ([10,20]) have also been successfully identified. The above studies highlight the potential of using local sheep breeds raised in different environments as a means to detect adaptive loci contributing to environmental adaptation.

Driven by the diversity of the ecological niches (variable topography, vegetation and climatic conditions) across the country, this study was primarily designed to identify plausible genetic loci associated with local adaptation (LA) in sheep. To this end, mainland sheep populations (Epirus and Peloponnesus) were compared to insular ones (Skyros, Lemnos, and Lesvos), assuming environmentally driven selection throughout: i) has been the primary driving force for LA, and ii) has affected the genomes of sheep raised in similar habitats in the same way.

In the present study, first we examined whether mainland-island present genetically different entities and then conducted genome scans and genome-wide association analysis in attempts to address two questions with regard to local adaptation (LA). Specifically: a) does LA leave detectable differential footprints in sheep genome? and b) can genetic differentiations lead to detection of functionally relevant genes to LA?

## Materials and methods

### Ethics statement

All applicable ethical guidelines for the care and use of animals were followed and animal blood samples were collected by trained personnel under strict veterinary rules. All samples and data in our study were collected under the consent of the sheep breeders. The protocol of the present study was approved by the Research Ethics and Ethics Committee of the Agricultural University of Athens (no 54/2020 and no 18/2021) according to the article 23 of law 4521/2018 of the Greek government.

### Data and quality control

A total number of n = 311 sheep blood samples were collected from 17 herds across 5 regions (3 island and 2 mainland) of Greece (see S1 Table and S1 Fig). All animals were genotyped with the Illumina Ovine 50K SNP array by Neogen Europe Ltd (Ayr, UK) and genotypes presented with a GenCall score greater than 0.30 were considered called. Quality control (QC) was performed at a sample and marker level. At the animal level, n = 12 samples were excluded due to call rate lower than 0.95. An additional number of 62 related animals were removed based on an identity by descent (IBD) degree of recent shared ancestry ($\hat{\pi }$) threshold of 0.25 (second-degree relatives). A $\hat{\pi }$ threshold higher than the typical one (0.1875 i.e. halfway between third- and second-degree relatives [21]) was used here to avoid potential bias due to cryptic relatedness and the removal of a large number of samples. At the marker level, n = 14,402 of the 53,295 original number of SNPs were excluded due to: call rate lower than 0.95, minor allele frequency (MAF) lower than 0.05, deviation from Hardy–Weinberg equilibrium (HWE) using a Fisher exact test p-value<10^−5^ and linkage disequilibrium (LD) r^2^ levels (r^2^>0.50, window size: 50 SNPs, increment: 5 SNPs) and autosomal mapped SNPs. Application of QC criteria at the marker level resulted in a total number of 38,893 autosomal mapped SNPs retained for further analyses. QC was performed using the SNP & Variation Suite software (version 8.9.0). The distribution of samples across regions is shown in S1 Table and S1 Fig.

### Detection of genetic structure and phylogeny analysis

Detection of genetic structure was assessed using various approaches. First, the genomic relationship matrix (GRM) between all pairs of individuals using genotypes of the 38,893 SNPs was calculated. Next, Principal Component Analyses (PCA) of animals’ genetic relationships was conducted and the first two PCs were plotted on the two dimensional space to resolve genetic patterns in the samples. The above analyses were performed using SNP & Variation Suite software (version 8.9.0).

Pairwise Reynold’s genetic distances between regions were automatically obtained by the hapFLK software (version 1.4, https://forge-dga.jouy.inra.fr/projects/hapflk, [22]) and they were graphically visualized using the NeighborNet graph in SplitsTree5 [23].

Admixture analysis (AA) was also applied to infer assignment probabilities for mainland or island membership for individual samples. To this end, the ADMIXTURE software [24] was employed with K-value equal to 2, assuming two ancestor populations i.e. mainland and island. Admixture results were graphically visualized using Distruct from CLUMPAK (http://clumpak.tau.ac.il/ [25]).

### Spatial point patterns analysis

A spatial point patterns analysis (SPPA) was performed using the elements of the first PC of animals’ genetic relationships as the events (observations) to investigate whether genomic relationships are distributed randomly or follow a specific pattern (indicative of structure) across the geographical landscape of sampling sites. A Gaussian kernel (or covariance function) was employed to fit the first-order intensity (number of observations per unit area) of a spatial point pattern using procedure SPP in SAS (ver.9.4). Spatial patterns were then resolved by constructing plots of the nearest neighbor distance and the smoothed kernel density of animals’ relationships on the geographical landscape with X and Y representing longitude and altitude of the sampling sites, respectively. The global Moran’s index (I) [26] and the Z score were finally estimated via the Variogram procedure in SAS (ver. 9.4) to test the null hypothesis of no spatial, positive or negative spatial auto-correlation for sample values.

### Estimation of LD scores and LD decay by distance

First, LD scores for each of the 38,893 markers using two maximum windows sizes of 500 kb and 1000 kb were calculated. Windows were always centered around the marker being scored, unless the window was at the beginning or the end of a region. This estimation was carried out for the island and mainland populations. Finally, the Wilcoxon Rank Sum test as implemented in procedure NPAR1WAY in SAS (ver.9.4) was applied to test the hypothesis that the two LD score distributions differ with respect to their medians.

Second, the decline rate of LD with inter-marker distance (in kb) within genes (using the OAR4.0 annotation) was estimated by fitting the following equation:

$$y_{i}=\frac{1}{\left(1+4\beta d_{i}\right)}+e_{i}$$

where *y_i_* is the estimated ${\hat{r}}^{2}$ after correcting for sampling error using the formula of Hill and Robertson [27] r^′2=(r^2−1N)(1−1N) with N twice the number of sampled individuals. Furthermore, *d_i_* is the inter-marker distance (in kb) for marker pair i, *β* is the coefficient that describes the decline rate of LD with distance (large values indicate low extent of LD) and *e_i_* is a random residual with *e_i_*~(0, s^2^). A maximum inter-marker distance of 2,500 kb was considered here. This analysis was carried out with procedure NLMIXED in SAS (ver. 9.4) using r^2^ values of the markers that had no negative or 0 values (because of correction for sample size). This selection resulted in a total number of 35,911 and 37,276 r^2^ values for mainland and island sheep, respectively. Estimation of LD scores and LD r^2^ values was performed with SNP & Variation Suite software (version 8.9.0).

### Detection of signatures of selection

Signatures of positive selection were detected using various approaches, as described below. First, the F_ST_ ([28,29]) statistic of genetic differentiation based on allele frequency differences between mainland-island populations was calculated for the 38,893 markers, via SNP & Variation Suite software (version 8.9.0). The negative F_ST_ values obtained for 14,214 SNPs were set to 0, since negative values have no biological interpretation. Raw F_ST_ values were then ranked and values exceeding the conservative 99.999^th^ percentile (0.3554) bound of the empirical F_ST_ distribution were considered as outliers. As individual F_ST_ values may be subject to genotyping errors, we also estimated genome-wide smoothed F_ST_ estimates, by averaging F_ST_ values across 500 kb windows. Windows (n = 383 out of 5,243) with less than four SNPs were discarded, which resulted in on an average of 7.82 (SD = 2.14) SNPs per window (minimum = 4, maximum = 16). Here, smoothed F_ST_ values exceeding the 99.999^th^ percentile (0.1357) bound were considered as outliers pointing to genomic regions under positive selection.

As a complementary approach to F_ST_, we used the FLK statistic [30] to detect regions of outlying differentiation between mainland and island populations. FLK is an extension of Lewontin and Krakauer (LK) test [31] which accounts for population size heterogeneity and for the hierarchical structure between populations. Specifically, FLK tests the neutrality of polymorphic markers by contrasting their allele frequencies in a set of populations against what would be expected under neutral evolution. Under the null hypothesis of no selection, a neighbor joining tree based on a matrix of Reynolds genetic distances is built and the length of branch is expected to be proportional to the amount of genetic drift in each population.

For each SNP, the FLK estimate and its p-value were obtained using the hapFLK software (version 1.4, https://forge-dga.jouy.inra.fr/projects/hapflk, [22]). As input for FLK analysis, we used a ped PLINK file that was constructed with all genotypes and two population identifiers i.e., mainland (n = 90) and island (n = 147) sheep populations. FLK analysis was run for all chromosomes using 20 expectation maximization iterations. This analysis was carried out with hapFLK software (version 1.4, https://forge-dga.jouy.inra.fr/projects/hapflk, [22]). To identify SS, FLK p-values were finally corrected for multiple comparisons using the false-discovery rate (FDR) method in R (http://www.r-project.org/). Here, a marker with FDR p-value below or equal to 0.1 was considered significant.

A Bayesian framework was additionally applied to evaluate whether each locus is affected by selection using a reversible-jump Markov chain Monte Carlo (MCMC) approach and the BayeScan 2.0 software (http://www.cmpg.unibe.ch/software/bayescan/, [32]). During this analysis, the following settings were specified: burn-in of 10,000 iterations followed by 50,000 iterations, a thinning interval of 10 and prior odds equal to 1000 for the neutral model. Following documentation [32], we used a posterior probability (prob) higher than 0.99 to define a marker under selection.

### Assessment of selective process

First, we estimated summary statistics of F_ST_, genetic diversity within (estimated as the average observed Heterozygozity (Ho) of the two populations) and between populations (calculated as difference of Ho between the two populations) in 1 Mb flanking genomic regions surrounding the F_ST_ outlier(s) marker. Then, for the three parameters, non-parametric loess smooth curves obtained via the LOESS procedure in SAS (ver.9.4) were estimated and plotted to detect characteristic patterns after ‘removing’ local noise. Finally, following Booker et al. [6], a visual examination of the characteristic patterns of the loess smooth curves was performed to determine the type of selective process (global adaptation, local adaptation, or genetic drift) that has occurred.

### Genome-Wide Association Analysis (GWAS)

A single locus mixed (additive) model GWAS using the EMMAX algorithm [33] was applied to detect significant associations between SNPs (fitted as fixed effect covariates) and the two origins coded as a binary dependent variable (0: island, 1: mainland). No other covariate (e.g., herd) was included in the model as these were confounded with the dependent variable. During this analysis, animals’ relationships were included in the mixed model as random effects via the GRM. Note that inclusion of the GRM has been shown to correct for possible population structure and stratification in the data [34]. Genome–wide significance of SNPs was declared using a FDR p-value lower than 0.05. Q-Q plots were used to analyze the extent to which the observed distribution of the test statistic followed the expected (null) distribution. This analysis along with the estimation of the genomic inflation factor (λ) was done to assess potential systematic bias due to population structure or to the analytical approach [35]. All above analyses were carried out with the SNP and Variation Suite v8.3.4 (Golden Helix, Inc. 2015).

### Detection of positional candidate genes and reported QTLs

First, we searched within 500kb downstream and upstream flanking regions of each significant marker for positional candidate genes using the NCBI database ([36]) and the Oar_v4.0 assembly ([37]). Then, the ids of the significant SNPs were submitted to the Variant Effect Predictor (VEP, https://www.ensembl.org/Tools/VEP, [38]) tool to search for published QTLs according to Oar_v3.1 assembly. VEP retrieves QTL information via connections with Animal QTL database (Animal QTLdb) and Online Mendelian Inheritance in Animals (OMIA) database for *Ovis aries*. Positions of QTLs were remapped from Oar_v3.1 to Oar_v4.0 assembly using the Genome Remapping Service from NCBI database [39].

### Functional enrichment analysis

Positional candidate genes were subject to Gene Ontology (GO) Biological Process (BP) enrichment analysis using the ClueGO V2.5.7 [40] plug-in in Cytoscape V3.7.2 (http://cytoscape.org/, [41]). During this analysis, the latest update (released: 05.03.2021) of GO BP annotations were used for the species of *Ovis aries*. Each time, the input genes were compared with a reference list of ovine genes using the following settings: minimum number of genes per BP = 1, minimum percentage of genes included in GO BP = 3%, minimum GO levels = 3, maximum GO levels = 8 and minimum kappa score for GO BP network connectivity = 0.4. Here, GO BPs with FDR p-values lower than 0.05 for the right-sided hypergeometric test were considered as significantly enriched. The CluePedia V1.5.7 [42] plug-in of Cytoscape(http://cytoscape.org/, [41]) was also used to illustrate the significantly enriched GO BPs with their involved genes.

## Results

### Genetic structure

**Fig 1:**
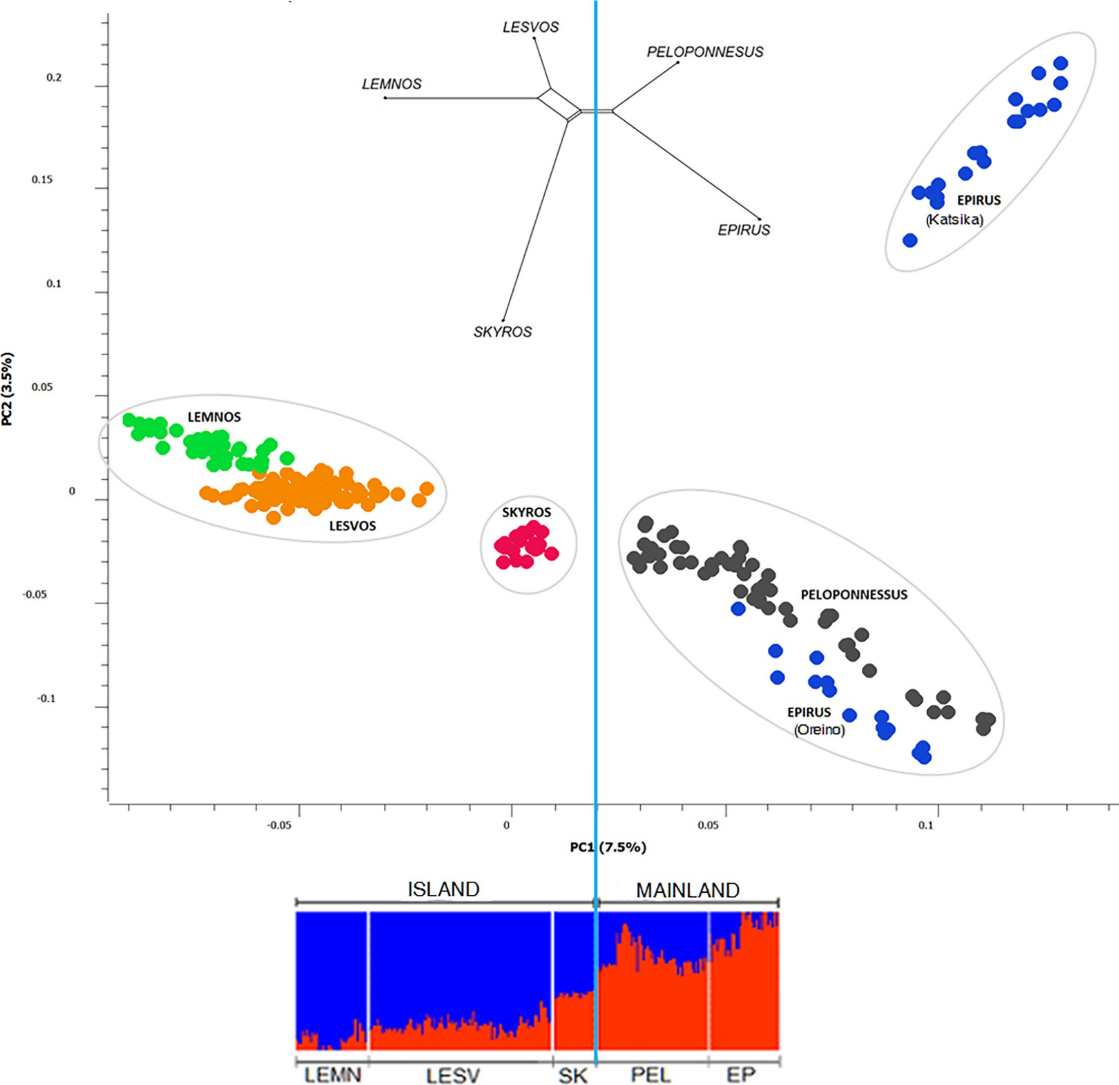
Two-dimensional plot of the first two PCs derived from PCA on animals’ genomic relationships. A total number of four clusters were created, one with genotypes from Lemnos (green) and Lesvos (brown) islands, a second with genotypes from Skyros island (red), a third with genotypes from Peloponnesus (dark grey) and Oreino breed of Epirus (blue) and finally a fourth with genotypes of Katsika breed of Epirus (blue). An unrooted phylogenetic tree depicting pairwise Reynold’s genetic distances between regions was also constructed (top). Individual assignment probabilities generated with ADMIXTURE (K = 2) are shown at the bottom of the graph. Each color represents a cluster, and the ratio of colored bars is proportional to the assignment probability of an individual to each cluster. Blue and red colors represent island and mainland membership, respectively. A possible partition of populations into mainland-island origin is indicated by the vertical blue colored line in the graph’s center.

The two-dimensional plot of the first two PCs derived from PCA on animals’ genomic relationships is shown in Fig 1. PCA revealed the formation of four clusters: one with Lemnos (green color) and Lesvos (brown color) samples, a second with genotypes from Skyros island (red) in the center of the plot, a third with genotypes from Peloponnesus (dark grey color) and the Oreino breed (Epirus, blue color) and finally a fourth cluster with genotypes from Katsika breed (Epirus, blue color, upper right). In the same plot, the first PC that accounted for most of the variation of animals’ relationships (7.5%) had the highest discriminatory power and separated island (Lemnos, Lesvos and Skyros) from mainland populations (Peloponnesus and Epirus). The second PC, which accounted for 3.5% of the variation of animals’ relationships, classified Epirus genotypes into two groups, each one corresponding to a different breed (Oreino and Katsika). In accordance with PCA results, the unrooted generated phylogenetic tree also suggested genetic discrimination of mainland-island genotypes with one branch containing Lemnos, Lesvos and Skyros samples and other samples from Peloponnesus and Epirus. At the bottom of Fig 1, the results of AA assuming two ancestral populations (K = 2) were consistent with the two previous analyses, showing a differential distribution of assignment probabilities of samples across origins with higher assignment probabilities of island membership (denoted as blue color) for samples of Lemnos, Lesvos and Skyros, and diminishing for samples of Peloponnesus and Epirus (Fig 1).

### Spatial point patterns analysis

**Fig 2:**
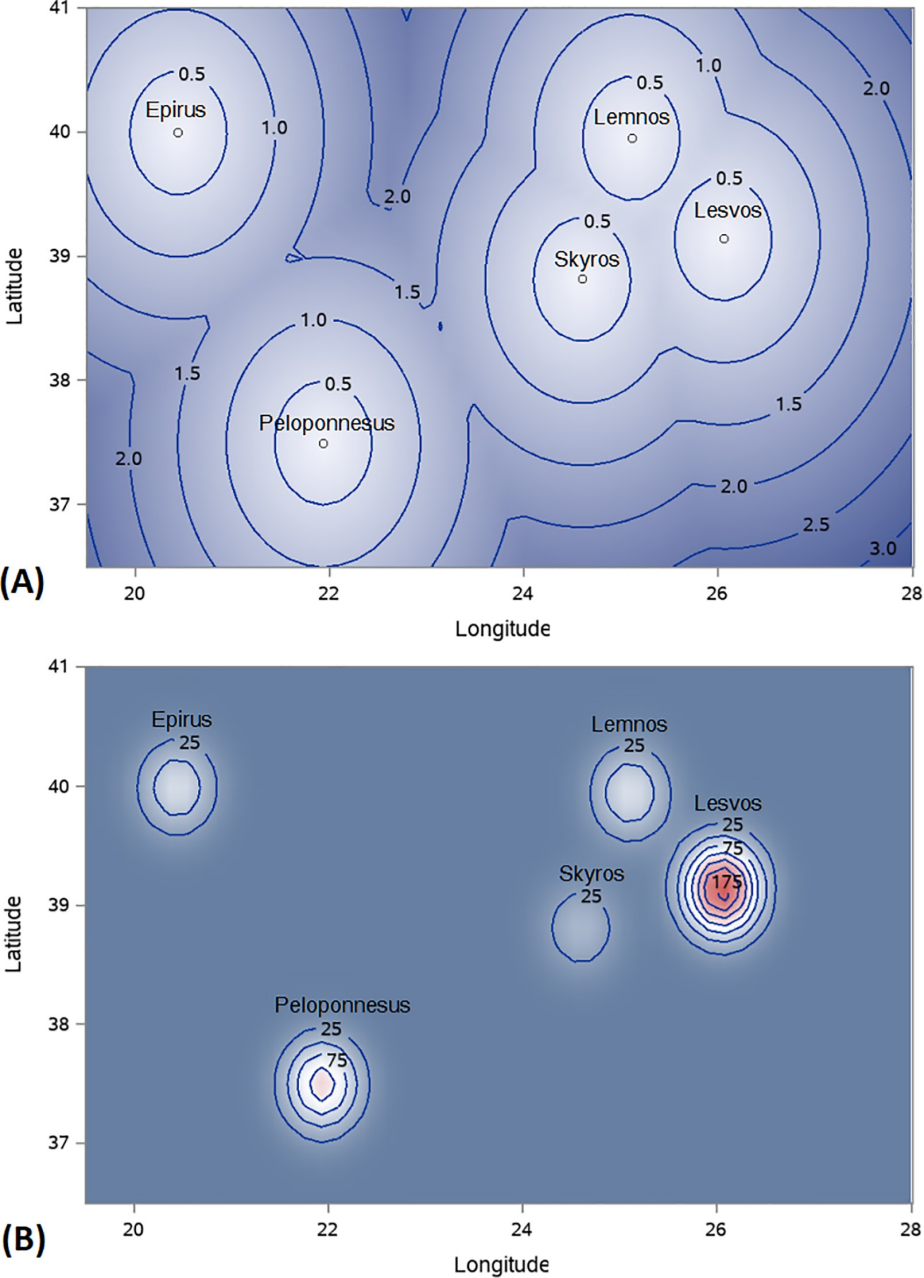
Plots of the mean nearest neighbor distances (A) and Gaussian kernel smoothed intensity (B) of animals’ genomic relationships along the geographical landscape. The plots indicate that animals sampled from the same location have higher genetic similarity than distantly placed or randomly selected samples.

Results of SPPA of animals’ genomic relationships (accounted here by the first PC) are depicted on Fig 2. As the nearest neighbor distance (Fig 2A) and the Gaussian kernel intensity (Fig 2B) plots portray, animals’ genomic relationships are not randomly distributed throughout the study area, and samples with similar values tend to cluster together according to geographical coordinates of sampling locations. In other words, animals sampled from the same location have greater genetic similarity than animals from distant locations or randomly selected. These results were confirmed by the estimated global Moran’s I index (I = 0.8012, p<0.0001) and the positive Z score (Z = 83.5) supporting the hypothesis of non-zero, positive spatial autocorrelation for genomic relationships, respectively (results not shown). Overall, these findings suggest that the five sheep populations are distinct genetic entities that can be further classified based on their origin, mainland vs. island, allowing for further investigation of their genomic characteristics.

### Estimation LD score and rate of LD decay by distance

**Fig 3:**
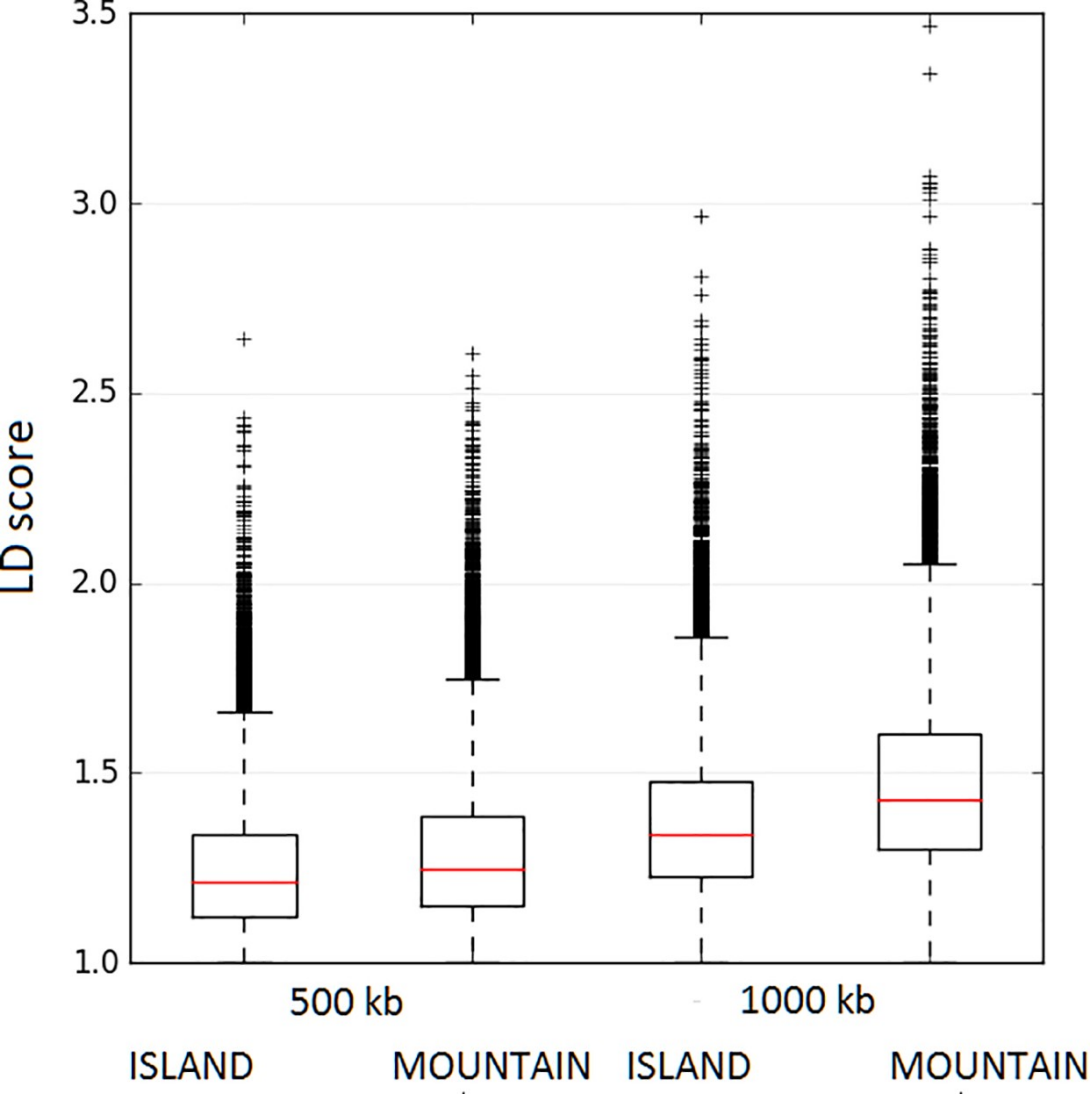
Box plots of LD scores for 500 kb and 1000 kb windows for mainland- island sheep populations.

Fig 3 shows Box plots of LD scores across the two origins for two windows sizes: 500 kb and 1000 kb. Median LD score was higher in the mainland vs. island sheep at 500 kb (1.2479 vs. 1.2103, p<0.001, Wilcoxon Two-Sample test) and the difference was more pronounced for the 1000 kb windows (1.4314 vs. 1.3363, p<0.001, Wilcoxon Two-Sample test). Results of LD score estimates were confirmed by *β* coefficient estimates obtained from nonlinear regression analysis with *β* estimate higher for island (is) (*β*_*is*_ = 0.1173 ± 0.000953, CI(95%) = 0.1154–0.1191 when contrasted to its respective counterpart for mainland (m) sheep (*β*_*m*_ = 0.1062 ± 0.000916, CI(95%) = 0.1044–0.1080) (results not shown). Note that as the CI(95%) of the two *β* estimates do not overlap, the two estimates are statistically significantly different. As *β* coefficients describe the decline rate of LD by distance, high and low *β* estimates imply low and high LD persistency, respectively. Thus, both LD score and *β* coefficient estimates indicate lower extent of LD (or lower LD persistency) for the island compared to mainland sheep.

### Signatures of selection and GWAS signal(s)

**Fig 4:**
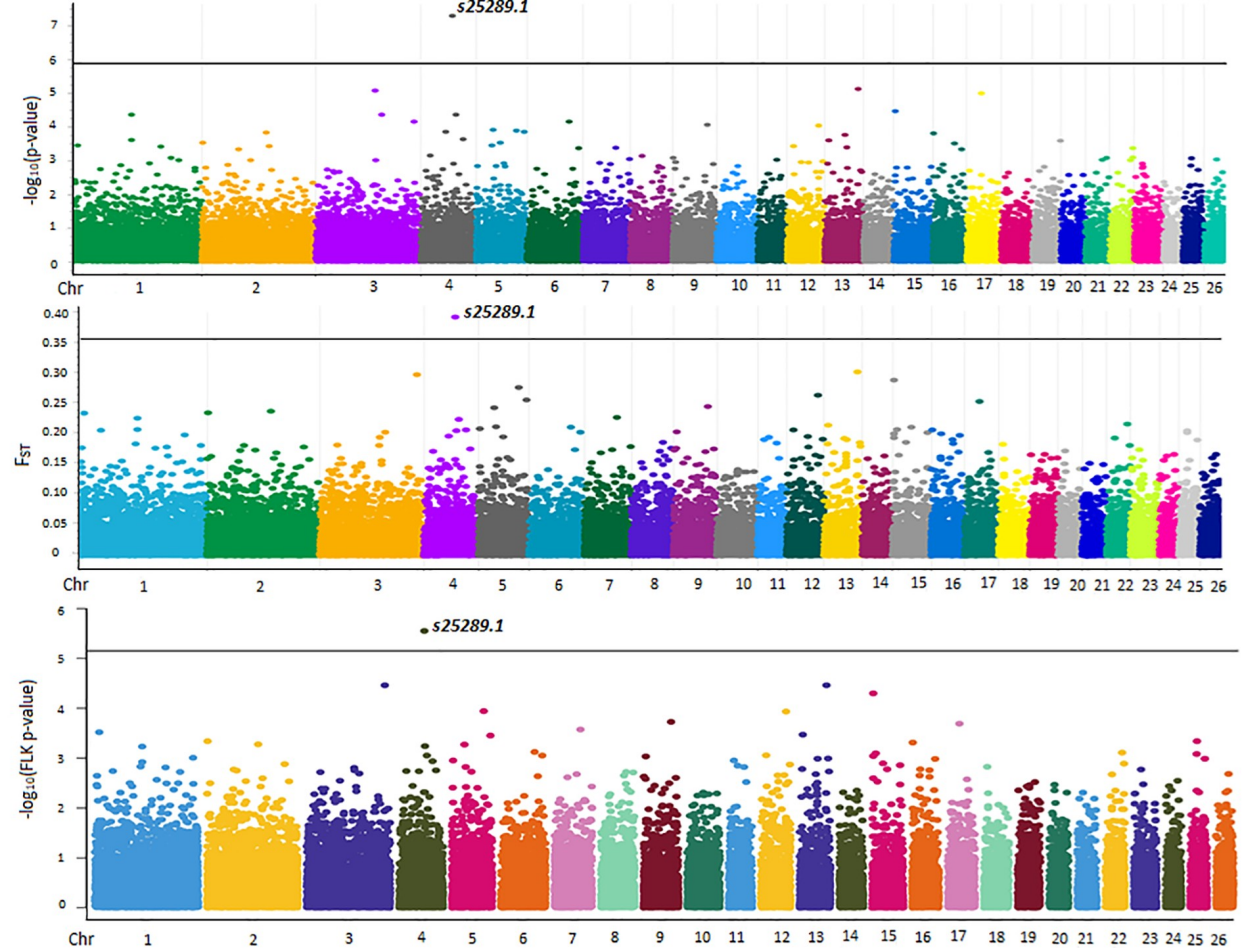
Manhattan plots of–log_10_(p-values) of SNPs (top), raw F_ST_ estimates (middle) and FLK statistic (bottom) across autosomes. Grey line on the upper Manhattan plot denote threshold (-log_10_(p-value) = 5.893) for genome wide significance, in the middle the 99.999^th^ percentile bound (0.3554) and the threshold for the FLK statistic (bottom). The id of significant SNP is also presented. FLK plot was constructed with the CMplot package (https://github.com/YinLiLin/R-CMplot) in R (http://www.r-project.org/).

Table 1 summarizes the outlier markers obtained from F_ST_ and FLK along with results of GWAS. Only one marker i.e. *s25289*.*1* (OAR4, position 72,769,785) had a raw F_ST_ value (0.3909), exceeding the 99.999^th^ bound (0.3554) (Fig 4). The same marker displayed a p_FDR_-value slightly higher than 0.10 for the FLK statistic (FLK p-value = 2.83e-06) and had a p_FDR_-value (0.002 and -log_10_(p-value) = 7.7260) lower than the 0.05 (-log_10_(p-value) = 5.890) threshold denoting genome wide significance during GWAS (Fig 4). With regard to the latter, the estimated λ value of the observed p-values (λ = 0.9870) along with the Q-Q plot of expected vs. observed SNP p-values denoted no population structure or other artifact(s) in the data (S2 Fig). With regard to smoothed F_ST_ estimates, only one window-based estimate (F_ST_ = 0.1382) exceeded the 99.999^th^ bound (0.1357) of the empirical window-based F_ST_ distribution and this window included marker *s25289*.*1*. Results of F_ST_ outlier(s) detection were also confirmed with Bayescan with a posterior probability (prob) equal to 0.9946 (p_FDR_-value = 0.005, F_ST_ = 0.18836, results not shown).

**Table 1:** Significant SNPs obtained by allele frequency-based F_ST_, FLK analysis and GWAS.

					GWAS				
Marker	OAR	Position	F_ST_ (raw)	FLK (p-value, p_FDR_)	p-value	-log_10_ (p-value)	p_FDR_	PVE (%)	Major allele (frequency)
*s25289*.*1*	4	72,769,785	0.3909	2.83e-06, 0.11	5.1482e-08	7.2883	0.002	0.1188	B (0.717)

### Evidence for local adaptation

**Fig 5:**
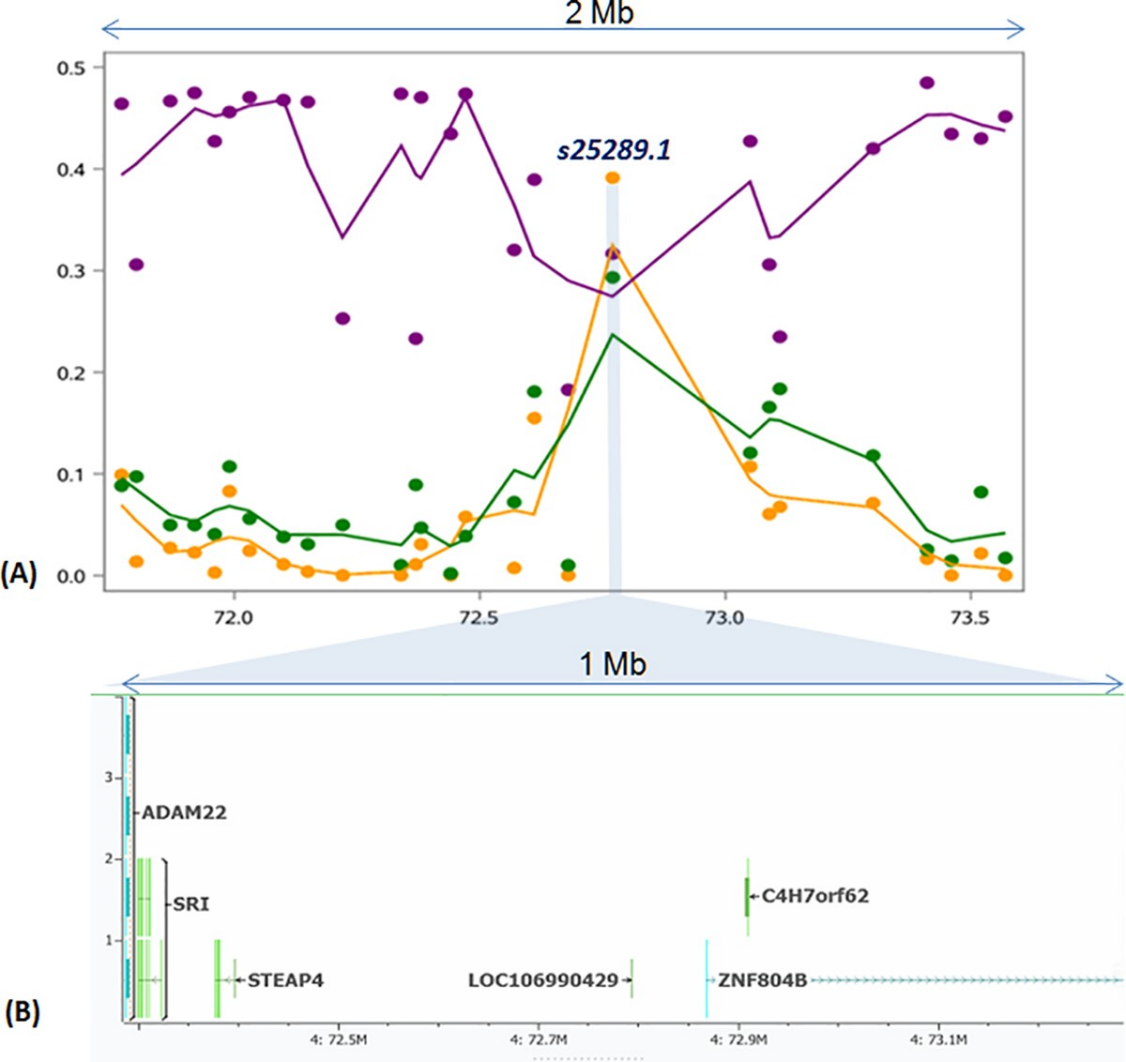
(A) Loess curves of F_ST_ (orange line), within diversity (purple line) and between diversity (green line) for 1 Mb genomic distances around the F_ST_ outlier marker (s25289.1). Smoothed lines were obtained via LOESS procedure in SAS (ver.9.4) using a smoothing parameter equal to 0.21 for the three variables and 27 points (markers) (B) Positional candidate genes within 500 kb down- and up-stream the significant marker (s25289.1) are also shown here.

Patterns of the three summary statistics (F_ST_, genetic diversity within (avHo) and between populations (dHo)) in 1 Mb genomic regions surrounding the F_ST_ outlier marker are shown in Fig 5A. As shown in Figure, the peak of F_ST_ values (orange line) at marker position is accompanied by a decrease in avHo (purple line) and a notable peak of dHo (green line). The latter, which is typical of long-term local adaptation, excludes the scenario of global adaptation and genetic drift ([6,51]). All of the above analyses indicate that marker *s25289*.*1* is in a genomic region containing genetic loci that are most likely involved in the long-term LA process.

### Positional candidate genes and published QTLs

A total number of 6 positional candidate genes i.e., 5 protein coding genes (*ZNF804B*, *C4H7orf62 or TEX47*, *STEAP4*, *SRI and ADAM22*) and one pseudogene (*LOC106990429*) were detected within the searched genomic areas around the significant marker (*s25289*.*1*) on OAR4 (Table 2 and Fig 5B). The nearest gene to the significant SNP was *LOC106990429* while *ADAM22* gene was located furthest away from the marker (494 kb). In addition, two published QTLs (ID126988, 4:66,776,665–70,273,454 and ID127002, 4:68,271,877–69,172,400) related to tail fat deposition are reported, distanced ~800 kb downstream the marker. Note that at a more distant location (~1.62 Mb) upstream the marker resides another member of the *STEAP* family genes, namely *STEAP2* (OAR4: 74,395,870–74,420,792).

**Table 2:** Positional candidate genes around the *s25289*.*1* significant marker on chromosome 4.

Gene ID	Gene description	Extent of gene (bp)^a^	Distance from marker (bp)
*LOC106990429*	*histone-lysine N-methyltransferase SETMAR-like*	72777738–72778750	8965
*ZNF804B*	*zinc finger protein 804B*	72852055–73433337	82270
*C4H7orf62 or TEX47*	*testis expressed 47*	72880869–72895751	125966
*STEAP4*	*STEAP4 metalloreductase*	72360288–72381700	388085
*SRI*	*sorcin*	72283566–72307498	462287
*ADAM22*	*ADAM metallopeptidase domain 22*	72158944–72275420	494365

^a^ Positions were based on Oar_v4.0 assembly according to NCBI database.

### Functional enrichment analysis

**Fig 6:**
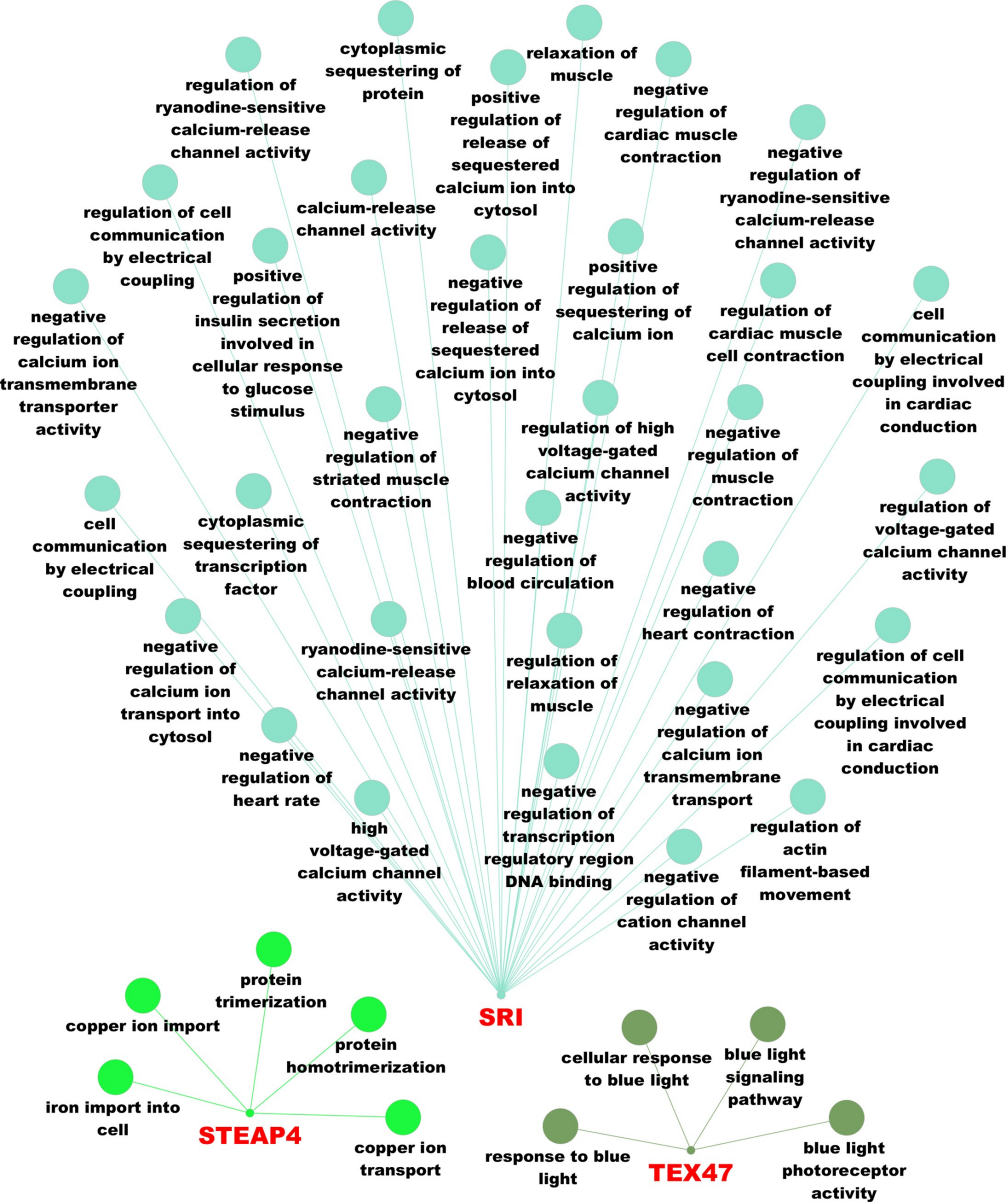
Three network representations of the total number of significantly enriched GO BP terms (nodes with khaki, light green and light blue color) and their respective member genes (red labels). Figure was constructed via ClueGO V2.5.7 and CluePedia V1.5.7 plugins in Cytoscape.

All genes were recognized by ClueGO. As seen in Fig 6, functional enrichment analysis resulted in 41 significantly enriched GO BPs with three member genes (*TEX47*, *STEAP4* and *SRI*). The maximum number of enriched GO BPs (n = 32) were detected for *SRI* gene while *TEX47* gene presented the minimum number of GO BPs (n = 4). With regard to the number of most significantly enriched GO BP terms, 3 GO BPs were revealed for *TEX47* gene while one GO BP was found for *STEAP4* and *SRI* genes (S2 Table). Specifically, 3 enriched GO BPs i.e., blue light signaling pathway (GO:0009785), cellular response to blue light (GO:0071483) and blue light photoreceptor activity (GO:0009882) presented the same low FDR p-value (0.006) for *TEX47* gene (S2 Table). For *STEAP4* gene, iron import into cell biological process (GO:0033212) showed the lowest FDR p-value (0.007) while the most significantly enriched GO BP for *SRI* gene was the negative regulation of cardiac muscle contraction (GO:0055118) with FDR p-value as high as 0.006.

## Discussion

### Different LD patterns in mainland—island sheep

The current study sought to answer the intriguing question of whether LA leaves distinct genome footprints manifested in terms of LD structure. The current findings provided an answer to this question, indicating that mainland sheep have higher LD persistency than island sheep. In both populations, LD decays fast that is typical for local breeds experiencing weak artificial selection in contrast to ‘commercial’ breeds where LD extends for larger genomic distance [43]. Given that the mating system for such breeds is relatively comparable across the country, the slight differences in LD decay between the two populations are most likely due to differences in effective population sizes. Yang et al. [44] have also observed overall slow decay rate and a high level of LD for the plateau (Tibetan and Yunnan–Kweichow) sheep breeds when compared to breeds from Northern and Eastern China the latter exhibiting a rapid decay rate and low LD levels. In buffaloes, different patterns for LD decay for swamp and their river counterparts are reported, with higher rates for LD decay in the swamp when compared to the river group [45]. In farmed Atlantic salmon, different LD patterns between populations adapted to different environments across hemispheres have been observed [46] with authors emphasizing that exposure to different management and environmental conditions over just a few generations be sufficient to generate large changes in the genetic structure of farmed salmon populations with the same genetic origin. In line with results for salmon, populations of common carp originating from different geographic regions from China showed distinct LD decay patterns with distance [47]. Since different LD patterns can emerge for a variety of reasons, including mating system, sample size, population background, and demography [48], it’s impossible to pinpoint and quantify each factor’s exact contribution.

### Geography or environmental related adaptation?

The genetic patterns discovered here corroborate the genetic similarity of Lemnos and Lesvos sheep, probably due to past gene flow [3]. Furthermore, they uncover the genetic uniqueness of the Skyros and the Katsika sheep from Epirus. Finally, they suggest a genetic relatedness between the Oreino (in Greek meaning mountainous) Epirus sheep with the mountainous Peloponnesus sheep. The latter suggests either a common ancestor for the two populations or past gene flow between Epirus and Peloponnesus mountainous sheep following possibly human population migration routes.

Intuitively, the genetic variation of the studied populations herein is interpreted in terms of environmental adaptation. Yet, as the studied populations are placed on an east-west geographic axis, genetic variation may equally fit a geographical axis instead of an environmental axis. In line with such a scenario, results of population structure and SPPA are supportive of an east-west geographical gradient with Skyros sheep positioned in the middle of the two geographical extremes (Lemnos—Lesvos and Epirus). As geography overlaps with climatic zones in the way that (east) insular sheep are kept on semi arid areas and (western) mainland sheep in rainy, humid and high altitude areas, it is impossible to identify the exact source of genetic differentiation. In general, regional origin has been identified as the main determinant of genetic differentiation of numerous European sheep breeds with three Greek breeds (Chios, Kymi and Lesvos) placed intermediate on an east–west cline between the fat-tailed Asian and European sheep [49]. Since the animals of the present study have been kept at their sampling sites for decades, we assess that genetic differentiation is more likely reflecting adaptation processes to local environmental conditions than possible regional differences.

### Evidence for long term local adaptation

Current results suggested that mainland sheep differed genetically from island sheep, with genetic differentiation reaching its peak at marker *s25289*.*1* on OAR4. Genomic regions with such outlier markers are intuitively interpreted as evidence of LA. Nonetheless, apart from LA, extreme F_ST_ outliers can also be generated by global adaptation and genetic drift [6]. Since different processes leave different footprints on the genomic landscape, examining the pattern of summary statistics, such as F_ST_, genetic variation within and between populations in genomic regions surrounding F_ST_ outliers, may aid in determining which type of selective processes has occurred ([6,50]). Following this rationale, the peak of dHo observed in Fig 5 conforms to long-term LA arising from spatially antagonistic pleiotropy ([6,51], Fig 3, case C). The importance of *s25289*.*1* was also confirmed by association analysis, raising the possibility that the surrounding genomic region contains one or more genes involved in long-term LA in sheep. We will discuss this issue in more detail in the next section.

### Functional candidate genes

Three of the candidate genes were associated with significantly enriched GO terms. Of these, *TEX47* is associated with responses to blue light, including photoreceptor activity. In animals, light in the range from 400 to 500 nm is sensed via blue and ultraviolet photoreceptors that are called cryptochromes. Cryptochromes act as components of the circadian clock that control daily physiological and behavioral rhythms and as photoreceptors that mediate entrainment of the circadian clock to light [52].

The next candidate gene i.e. *SRI* was found to be involved in multiple BPs including positive regulation of insulin secretion involved in cellular response to glucose stimulus, excitation-contraction coupling in the heart, calcium homeostasis in the heart sarcoplasmic reticulum and modulation of the activity of RYR2 calcium channels [53].

With regard to *STEAP4*, associated GO BP terms revealed its involvement in iron and copper import/transport that is critical to the maintenance of cellular homeostasis [54]. In humans, the gene has been linked to obesity [55–57], while in sheep, *STEAP4* [58] and other members of the same gene family (*STEAP3)* have been associated with excess fat accretion in tails [59]. The latter is a valuable energy reserve, playing a particular role in adaptation to harsh conditions (e.g. drought seasons, extreme cold winters and food shortages) allowing fuel reserves to be mobilized when underfed and restored during the more favorable season [60]. Apart from the regulatory circuits of fat storage and mobilization in white adipocytes, the adipose tissue provides a unique thermogenic mechanism (via the brown and beige adipocytes) to maintain euthermia in response to cold exposure ([61,62]) that is critical for the survival of the newborns [63]. Finally, the adipose tissue acts as an endocrine organ and produces numerous bioactive factors such as adipokines and lipokines that communicate with other organs and modulate a range of metabolic pathways [64]. All of the above points to the adipose tissue’s role in the adaptive regulation of systemic metabolic homeostasis in response to nutrient excess or fasting, cold exposure [62], or physiological status.

With regard to rest genes with no detected enriched GO terms, *ADAM22* is a member of the ADAM (A Disintegrin And Metalloprotease) gene family that are involved in a broad range of biological processes such as cell fusion, spermatogenesis and development [65] and in the regulation of cell adhesion and inhibition of cell proliferation [66]. The gene has been associated with rumen microbial composition in sheep [67] and with reproductive traits in Nelore cattle [68].

Finally, *ZNF804B* is found to be located in genomic regions with overlapping SS and Copy Number Variations that were associated with selective adaptation in cattle [69].

## Conclusions

In conclusion, here we report a clear genetic differentiation of mainland vs. island Greek indigenous breeds that can be explained either by longitudinal or environmental factors or both. Furthermore, a genomic region (OAR4: 72.4–73.3Mb) associated with LA was identified. Consistently with previous findings [10], this region is likely to be important for local adaptation in sheep and harbors five functionally relevant genes, *TEX47*, *STEAP4*, *SRI*, *ADAM22 and ZNF804B*, that warrant further investigation.

## Supporting information

S1 Fig Map of sampling locations of sheep herds in Greece. (TIF)

S2 Fig Quantile- quantile (Q-Q) plot of GWAS results for the two origins (island vs. mainland). Blue dots denote the −log_10_(p-value) obtained from the single locus mixed model and the red line represents the expected values for the null hypothesis under no association.
(TIF)

S1 Table Distribution of samples per origin and region within origin (n: Number of samples per region). (DOCX)

S2 Table Results by Gene Ontology biological process (GO BP) enrichment analysis. (DOCX)
