# Supplementary figures and images for: Genetic differentiation of mainland-island sheep of Greece: Implications for identifying candidate genes for long-term local adaptation
Source: PLoS One. 2021 Sep 16;16(9):e0257461. doi: 10.1371/journal.pone.0257461 (PMC8445479; doi:10.1371/journal.pone.0257461)

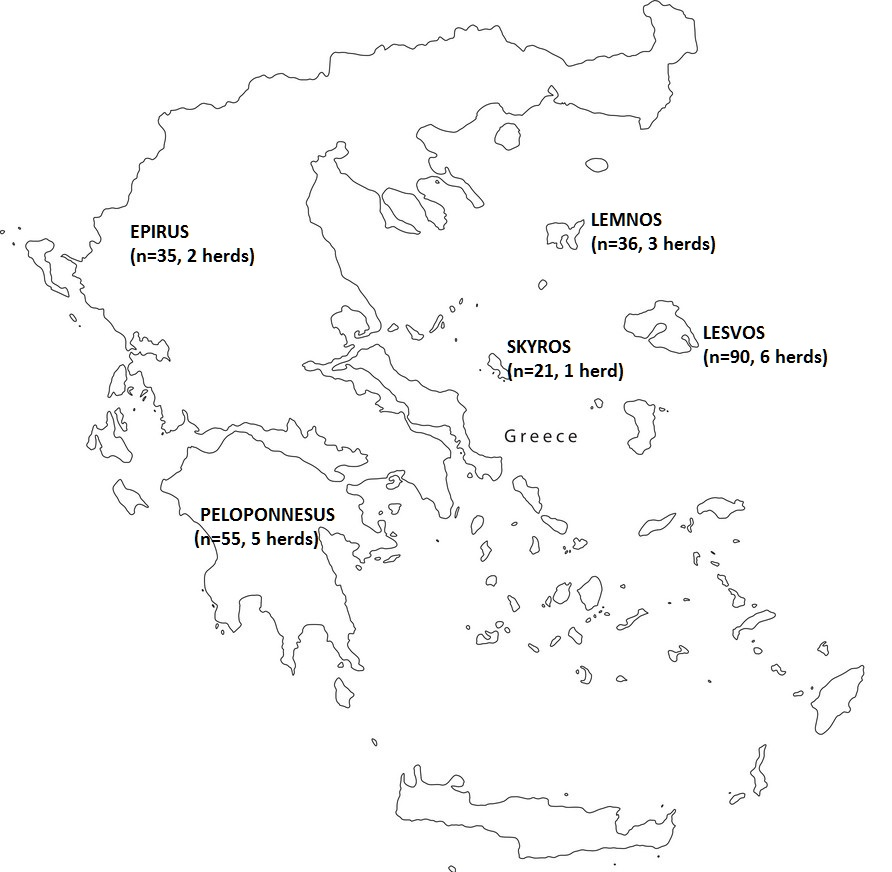

Supplement: S1 Fig — (TIF) [file pone.0257461.s001.tif]

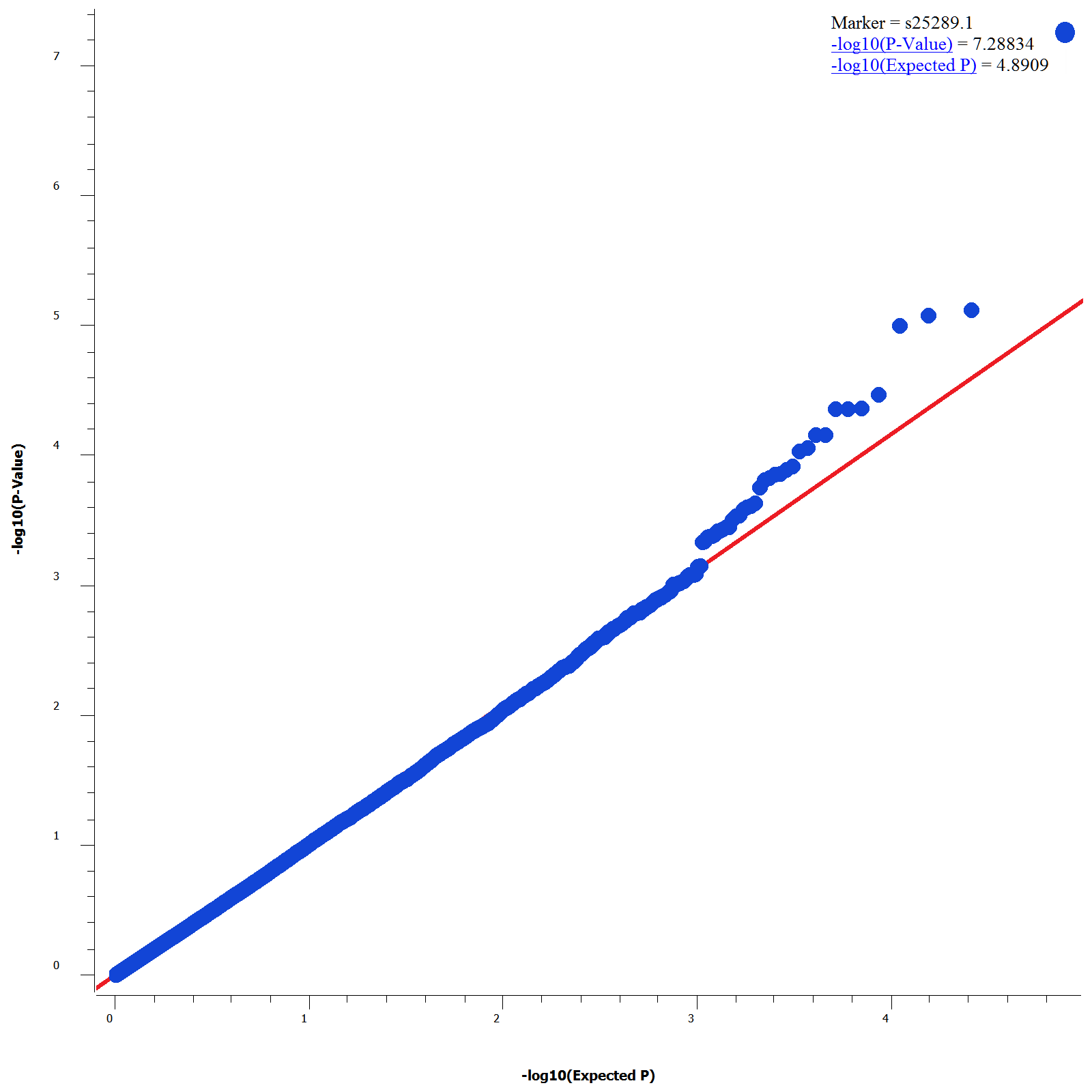

Supplement: S2 Fig — Blue dots denote the −log10(p-value) obtained from the single locus mixed model and the red line represents the expected values for the null hypothesis under no association. (TIF) [file pone.0257461.s002.tif]
