# Supplementary material for: Genetic differentiation of mainland-island sheep of Greece: Implications for identifying candidate genes for long-term local adaptation
Source: PLoS One. 2021 Sep 16;16(9):e0257461. doi: 10.1371/journal.pone.0257461 (PMC8445479; doi:10.1371/journal.pone.0257461)
Supplement: S1 Table — (DOCX) [file pone.0257461.s003.docx]

S1 Table. Distribution of samples per origin and region within origin (n: number of samples per region).

| **Origin** | **Region** | **Latitude** | **Longitude** | **No of herds/**  **no of samples per herd (in parentheses)** | **n** |
| --- | --- | --- | --- | --- | --- |
| **Island (n=147)** | \| **Lemnos** \| \| --- \| | 39.9535 | 25.1098 | Herd1 (11), Herd2 (14), Herd3 (11) | **36** |
|  | **Lesvos** | 39.1424 | 26.0576 | Herd1 (17), Herd2(18), Herd3(13),  Herd4(14), Herd5 (14), Herd6 (14) | **90** |
|  | **Skyros** | 38.8160 | 24.6032 | Herd1 | **21** |
| **Mainland (n=90)** | **Epirus** | 39.9973 | 20.4411 | Herd1 (19), Herd2 (16) | **35** |
|  | **Peloponnesus** | 37.4965 | 21.9350 | Herd1 (8), Herd2 (12), Herd3 (10),  Herd4 (16), Herd5 (9) | **55** |
|  | **Total** |  |  |  | **237** |
