# Supplementary material for: Genetic differentiation of mainland-island sheep of Greece: Implications for identifying candidate genes for long-term local adaptation
Source: PLoS One. 2021 Sep 16;16(9):e0257461. doi: 10.1371/journal.pone.0257461 (PMC8445479; doi:10.1371/journal.pone.0257461)
Supplement: S2 Table — (DOCX) [file pone.0257461.s004.docx]

| S2 Table. Results by Gene Ontology biological process (GO BP) enrichment analysis. | | | |  |
| --- | --- | --- | --- | --- |
|  |  |  |  |  |
| **GO BP term** | **GO ID** | **P-value** | **FDR p-value** | **Gene** |
| response to blue light | GO:0009637 | 0.001288193 | 0.008158554 | *TEX47* |
| blue light signaling pathway | GO:0009785 | 0.000322165 | 0.006121134 | *TEX47* |
| cellular response to blue light | GO:0071483 | 0.000322165 | 0.006121134 | *TEX47* |
| blue light photoreceptor activity | GO:0009882 | 0.000322165 | 0.006121134 | *TEX47* |
| copper ion transport | GO:0006825 | 0.003539535 | 0.007472351 | *STEAP4* |
| protein trimerization | GO:0070206 | 0.003539535 | 0.007472351 | *STEAP4* |
| iron import into cell | GO:0033212 | 0.001931822 | 0.007340924 | *STEAP4* |
| copper ion import | GO:0015677 | 0.001288193 | 0.008158554 | *STEAP4* |
| protein homotrimerization | GO:0070207 | 0.002896683 | 0.007862425 | *STEAP4* |
| calcium-release channel activity | GO:0015278 | 0.00739012 | 0.007800682 | *SRI* |
| negative regulation of release of sequestered calcium ion into cytosol | GO:0051280 | 0.001931822 | 0.007340924 | *SRI* |
| positive regulation of release of sequestered calcium ion into cytosol | GO:0051281 | 0.007710498 | 0.007710498 | *SRI* |
| regulation of voltage-gated calcium channel activity | GO:1901385 | 0.003860844 | 0.007335604 | *SRI* |
| cytoplasmic sequestering of transcription factor | GO:0042994 | 0.001931822 | 0.007340924 | *SRI* |
| high voltage-gated calcium channel activity | GO:0008331 | 0.003218148 | 0.007643101 | *SRI* |
| ryanodine-sensitive calcium-release channel activity | GO:0005219 | 0.004824306 | 0.007638484 | *SRI* |
| regulation of ryanodine-sensitive calcium-release channel activity | GO:0060314 | 0.003860844 | 0.007335604 | *SRI* |
| regulation of high voltage-gated calcium channel activity | GO:1901841 | 0.00225352 | 0.007136147 | *SRI* |
| negative regulation of ryanodine-sensitive calcium-release channel activity | GO:0060315 | 0.001931822 | 0.007340924 | *SRI* |
| cell communication by electrical coupling | GO:0010644 | 0.004182076 | 0.007223585 | *SRI* |
| relaxation of muscle | GO:0090075 | 0.006107834 | 0.00773659 | *SRI* |
| regulation of cell communication by electrical coupling | GO:0010649 | 0.001610046 | 0.00764772 | *SRI* |
| cytoplasmic sequestering of protein | GO:0051220 | 0.004182076 | 0.007223585 | *SRI* |
| regulation of actin filament-based movement | GO:1903115 | 0.007069665 | 0.00790139 | *SRI* |
| negative regulation of blood circulation | GO:1903523 | 0.003860844 | 0.007335604 | *SRI* |
| negative regulation of muscle contraction | GO:0045932 | 0.003860844 | 0.007335604 | *SRI* |
| negative regulation of heart contraction | GO:0045822 | 0.003539535 | 0.007472351 | *SRI* |
| regulation of relaxation of muscle | GO:1901077 | 0.00225352 | 0.007136147 | *SRI* |
| negative regulation of heart rate | GO:0010459 | 0.001610046 | 0.00764772 | *SRI* |
| negative regulation of striated muscle contraction | GO:0045988 | 0.001288193 | 0.008158554 | *SRI* |
| negative regulation of transcription regulatory region DNA binding | GO:2000678 | 0.003539535 | 0.007472351 | *SRI* |
| cell communication by electrical coupling involved in cardiac conduction | GO:0086064 | 0.00225352 | 0.007136147 | *SRI* |
| negative regulation of cardiac muscle contraction | GO:0055118 | 0.000644252 | 0.006120394 | *SRI* |
| regulation of cardiac muscle cell contraction | GO:0086004 | 0.006428522 | 0.00763387 | *SRI* |
| positive regulation of sequestering of calcium ion | GO:0051284 | 0.00225352 | 0.007136147 | *SRI* |
| regulation of cell communication by electrical coupling involved in cardiac conduction | GO:1901844 | 0.001288193 | 0.008158554 | *SRI* |
| negative regulation of calcium ion transmembrane transport | GO:1903170 | 0.005145304 | 0.00752006 | *SRI* |
| negative regulation of cation channel activity | GO:2001258 | 0.007069665 | 0.00790139 | *SRI* |
| negative regulation of calcium ion transport into cytosol | GO:0010523 | 0.002896683 | 0.007862425 | *SRI* |
| negative regulation of calcium ion transmembrane transporter activity | GO:1901020 | 0.004824306 | 0.007638484 | *SRI* |
| positive regulation of insulin secretion involved in cellular response to glucose stimulus | GO:0035774 | 0.005787069 | 0.007853879 | *SRI* |
